# A modified isooctane‐based DNA extraction method from crude oil

**DOI:** 10.1002/mlf2.12081

**Published:** 2023-09-28

**Authors:** Armando Alibrandi, Rolando di Primio, Alexander Bartholomäus, Jens Kallmeyer

**Affiliations:** ^1^ GFZ German Research Centre for Geoscience, Section Geomicrobiology Potsdam Germany; ^2^ Aker BP Lysaker Norway

**Keywords:** crude oil, DNA extraction method, oil reservoir microbiome

## Abstract

Microbes from oil reservoirs shape petroleum composition through processes such as biodegradation or souring. Such processes are considered economically detrimental and might pose health and safety hazards. It is therefore crucial to understand the composition of a reservoir's microbial community and its metabolic capabilities. However, such analyses are hindered by difficulties in extracting DNA from such complex fluids as crude oil. Here, we present a novel DNA extraction method from oils with a wide American Petroleum Institute (API) gravity (density) range. We investigated the ability to extract cells from oils with different solvents and surfactants, the latter both nonionic and ionic. Furthermore, we evaluated three DNA extraction methods. Overall, the best DNA yields and the highest number of 16S rRNA reads were achieved with isooctane as a solvent, followed by an ionic surfactant treatment using sodium dodecyl sulfate and DNA extraction using the PowerSoil Pro Kit (Qiagen). The final method was then applied to various oils from oil reservoirs collected in aseptic conditions. Despite the expected low cell density of 10^1^–10^3^ cells/ml, the new method yielded reliable results, with average 16S rRNA sequencing reads in the order of 41431 (±8860) per sample. Thermophilic, halophilic, and anaerobic taxa, which are most likely to be indigenous to the oil reservoir, were found in all samples. API gravity and DNA yield, despite the sufficient DNA obtained, did not show a correlation.

## Impact statement

The scope of this work was to systematically assess how to extract DNA from crude oil. We carried out a series of assessments of previously suggested reagents and protocols to identify the most suitable reagents and procedures for every step of the process. This study provides a method that can be applied to oils of a wide compositional range.

## INTRODUCTION

It has long been suspected that microbial communities exist in oil reservoirs^1^. However, a body of convergent observations demonstrating the existence of indigenous microbes in oil has only evolved in the last three decades^2^.

Microbial communities often shape their environment^3^, and oil reservoirs are no exception. Phenomena such as oil souring and oil degradation are mainly driven by microbial activity^2^, ^4^, ^5^, ^6^. Oil biodegradation is a process where the physical properties of the oil are changed via the selective metabolization of amenable hydrocarbon compounds or compound classes; biodegraded oils have a viscosity in the range of 1–10 million centipoise as compared to 10 centipoise or lower found in light oils^7^. Furthermore, biodegraded oils contain up to 5% sulfur by weight and are richer in resins, asphaltenes, and metals, while light oils have sulfur contents as low as 0.01% and are richer in paraffins with a high hydrogen‐to‐carbon ratio^8^. Density, in oil industry terms, is commonly described as American Petroleum Institute (API) gravity. The higher the density, the higher the level of biodegradation, and the lower the API gravity. API gravity is a dimensionless value, where 10 corresponds to water density. Extra heavy oils, such as tar, have an API gravity lower than 10 and sink in water. Heavy oils have API gravities in the range 10–20, medium oils have API gravities in the range 20–30, and everything above 30 is considered light crude.

Microbial oil souring, that is, the production of hydrogen sulfide (H_2_S) by sulfate‐reducing bacteria, causes multiple detrimental effects, ranging from pipe corrosion to health hazards due to H_2_S toxicity. Generally, biodegraded oils have lower economic value due to the difficulty they pose to refineries in processing caused by the higher sulfur and resin/asphaltene contents^9^.

It is therefore crucial to comprehend the composition and the activity of the indigenous microbial community in oil reservoirs to understand and possibly prevent deleterious microbial processes. Furthermore, investigating DNA found in oil can provide information on the overall life present in the reservoir. Specific DNA signals recovered from the reservoir could potentially be used as a marker to identify the source rocks, whether the oil has originated from one or multiple sources, and might provide information about its migration history.

Past efforts to identify microorganisms in oil reservoirs were mostly based on cultivation in growth media^10^, ^11^, ^12^. However, the unculturable portion of environmental microbes is around 90%–99%^13^ and it is reasonable to assume that this holds true for oil reservoirs as well^14^. To understand microbial reservoir dynamics, it is imperative to identify the taxa with cultivation‐independent methods.

According to the reviews from Wentzel et al. and Head^15^, ^16^, the considered indigenous taxa commonly found in high‐temperature (≥50°C) oil reservoirs are the phyla *Thermotogae*, represented by the genera *Petrotoga, Kosmotoga, Thermotoga, Geotoga, Oceanotoga*, and *Thermosipho*, and *Firmicutes*, with the genera *Thermoanaerobacter, Geobacillus, Bacillus, Desulfotomaculum, Caldanaerobacter*, and *Mahella*. Isolates from other bacterial groups are represented by the genera *Deferribacter* (*Deferribacteres*), *Thermus* (*Deinococcus‐Thermus*), *Anaerobaculum*, and *Thermovirga* (both Synergistetes). As archaeal isolates from high‐temperature oil reservoirs, the genera *Methanoculleus* and *Methermicoccus* (both *Methanomicrobia*), *Methanothermobacter* (*Methanobacteria*), *Methanococcus* (*Methanococci*), *Archaeoglobus* (*Archaeoglobi*), and *Thermococcus* (*Thermococci*) were found.

Modern molecular biological tools such as metagenomics and 16S rRNA sequencing offer a clearer view of the “inhabitants” of an ecosystem and, in the case of metagenomics, it is possible to get an impression of the microbial community's metabolic potential. These molecular biology tools require the extraction of the genetic material, that is, DNA, present in the oils.

Crude oil, however, is a complex matrix composed of a multitude of hydrophobic hydrocarbons^17^. The oil's complexity and its hydrophobicity make extraction of DNA, a hydrophilic molecule^18^, particularly challenging.

DNA itself is an easily accessible nutrient for microbial cells^19^, ^20^. Vuillemin et al.^21^ noted that extracellular DNA (eDNA) concentrations in sediments decrease rapidly with depth. Ramirez et al.^22^ reported that wherever there is a high abundance of eDNA, there is little to no compositional difference compared to the prokaryotic community found through sequencing. We, therefore, assume that most DNA found in crude oils is a truthful representation of the microbial community and that eDNA contribution is negligible. To obtain DNA, it is therefore required to disperse the oil matrix that contains the microbial cells holding the genetic material. To do so, multiple methods of cell separation from crude oil and oily sediments have been proposed. These methods can be categorized as solvent‐based methods and surfactant‐based methods.

Among the solvent‐based methods, Lappé and Kallmeyer^23^ suggested methanol or *n*‐hexane for the separation of cells from oily sediments, depending on the level of degradation of the oil. However, they were only interested in separating the cells for subsequent enumeration, not for downstream molecular analyses. Isooctane (2,2,4‐trimethylpentane) has also been suggested as a suitable solvent for cell extractions from oils and oily sediments^24^, ^25^, ^26^. Isooctane, together with *n*‐hexane, is one of the few solvents dissolving exclusively hydrophobic compounds^27^ but leaving hydrophilic substances, such as DNA^18^, intact. Methanol, on the contrary, cannot be a good candidate for DNA extraction as it also dissolves hydrophilic substances^28^. Isooctane and *n*‐hexane are therefore the best candidates for our purpose.

Surfactants, or surface‐active substances, are a class of compounds that have a hydrophilic and a hydrophobic group in the same molecule. Surfactants are capable of lowering surface tension and, in some cases, act as a detergent or emulsifier^29^. Detergents are a category of surfactants allowing the dispersion of water‐insoluble compounds in aqueous media, while emulsifiers are surfactants capable of counteracting droplet enlargement of the oily and aqueous phase, resulting in a uniform mixture of the two phases^30^.

There are two types of surfactants: ionic and nonionic. Typical representatives for nonionic surfactants are Span 80 and Tween 80^31^, whereas sodium dodecyl sulfate (SDS) is a typical representative of an ionic surfactant. The two types of surfactants act in different ways. Nonionic surfactants are generally gentler, do not interact with proteins, and, at low concentrations, they generally do not lyse cells^32^. Ionic surfactants tend to be harsher as they disrupt noncovalent bonds, they lyse cells, and, in the case of SDS, they have protein‐denaturing properties. They are also a detergent allowing the dispersion of water‐insoluble compounds in aqueous media^33^.

Surfactants consist of a molecule that combines both hydrophilic and lipophilic groups; for nonionic surfactants, the strength of these two opposing groups can be defined as the hydrophilic–lipophilic balance (HLB)^34^. HLB is expressed in an arbitrary scale from 1 to 20, where lower numbers correspond to more lipophilic surfactants and higher numbers correspond to hydrophilic ones. To obtain a successful emulsion with nonionic surfactants, a lipophilic and a hydrophilic surfactant, such as Span 80 and Tween 80, are used in combination. The ratio of the mix depends on the desired HLB value and varies depending on the types of compounds to be emulsified^31^. By changing the ratio of the surfactant mix and therefore the HLB value, it is possible to change the surfactant properties from that of an emulsifier to a wetting agent or an antifoaming agent for instance.

Surfactants suggested for DNA extraction from crude oil were solutions of hydrophilic Tween 20^24^ or Tween 80^35^ dissolved in phosphate buffer. The surfactant solutions had to be incubated overnight in a 1:1 ratio (oil/surfactant solution) at 50°C. This approach excluded the possibility of using combinations of solvents and surfactants as the solvent treatment requires overnight storage in a 1:1 solvent‐to‐oil ratio at 4°C^26^.

To our knowledge, no systematic DNA extraction method for crude oil samples has been published so far. Yoshida et al.^26^ were the first, to our knowledge, to publish a method for DNA extraction from petroleum. However, the focus of Yoshida et al.^26^ was not to assess the method but rather to discuss the content of the samples, and the actual method description was rather rudimentary.

Here, we describe a novel method for DNA extraction from crude oil that combines parts of previously published techniques with new methodological advances.

## RESULTS AND ASSESSMENT

**Figure 1:**
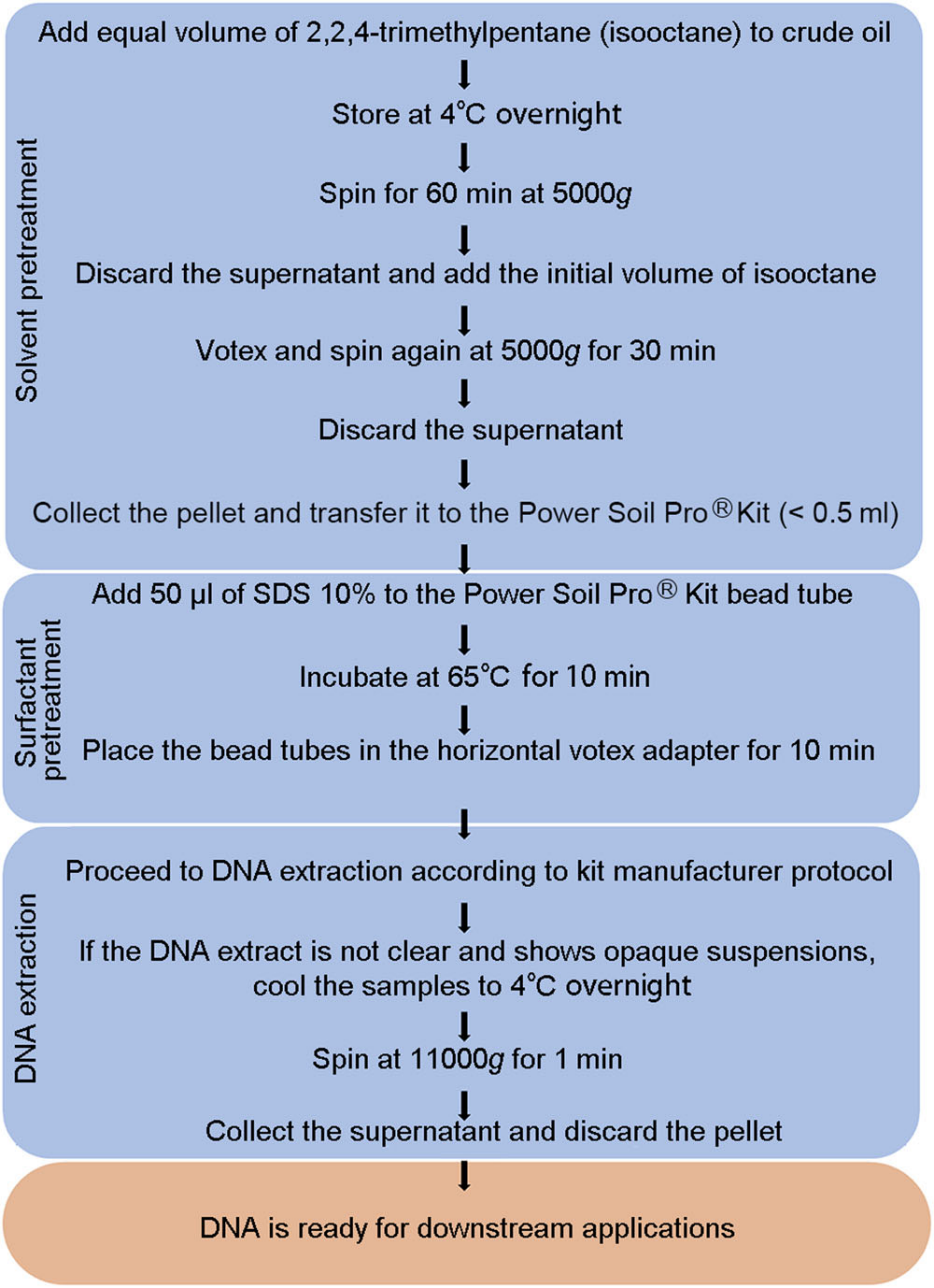
Flowchart showing the final method to obtain DNA from crude oil. Separated in blue blocks are the steps involving the solvent pretreatment, the surfactant pretreatment, and the DNA extraction.

We assessed the efficiency of the various methodological approaches through a series of experiments on test oils of different API gravities (T1, T2, T3, and T4). For each of the different steps of the overall extraction procedure, we identified the optimal methodology (Figure 1) that delivered the highest DNA yield and 16S rRNA sequencing reads. We afterward applied the developed method to pristine oils (P1, P2, and P3) originating from different oil reservoirs.

We separated the method into three different stages.

1. Solvent pretreatment.
2. Surfactant pretreatment.
3. DNA extraction.

The sequence of the stages was selected based on the order necessary to extract the DNA from the crude oil. A solvent step is required to reduce the volume of the sample and to obtain a pellet that can be inserted into the DNA extraction bead tube. Therefore, the solvent pretreatment is the initial stage. Because the surfactant treatment is not efficient on bulk oil samples, it has to be carried out after the solvent step. The surfactant pretreatment appeared to be effective in removing hydrocarbons and thereby further concentrating the DNA before the bead‐beating step.

We assessed the efficiency of the different treatments in four different experiments (Table 1) and gradually evaluated the best solvent, surfactant, and DNA extraction method.

**Table 1:** Summary of the experiments conducted to identify the optimal procedure for DNA separation from crude oil.

Experiment	Research objective	Method/reagent tested
Solvent test	Identify which solvent provides the highest DNA yield	*n*‐Hexane, isooctane
Surfactant test	Identify which surfactant provides the highest DNA yield	Tween 80/Span 80, SDS
Extraction test	Identify which extraction method provides the highest DNA yield	Norgen Olive Oil Kit, DNeasy PowerSoil Pro Kit (Qiagen), phenol–chloroform
16S rRNA sequencing	Identify which DNA extraction method provides the highest number of reads and determine the differences in microbial community composition between the extraction methods	

Each experiment was performed to address a specific objective.

The tests were executed in the order shown in Table 1. The treatment that resulted in the highest DNA yield was chosen and used in the next test.

To assess which solvent and surfactant are the most suitable, we needed a DNA extraction method known to extract measurable amounts of DNA from crude oil. Based on the available literature^26^, ^36^ and preliminary work, we designed an initial protocol using the DNeasy PowerSoil Pro Kit (Qiagen) for DNA extraction and isooctane as a solvent.

We hypothesize that there is a correlation between API gravity and DNA yields. The higher the level of biodegradation (i.e., the lower the API gravity), the higher the expected abundance of microorganisms in the oil and therefore the amount of extracted DNA should be.

### Solvent test

**Figure 2:**
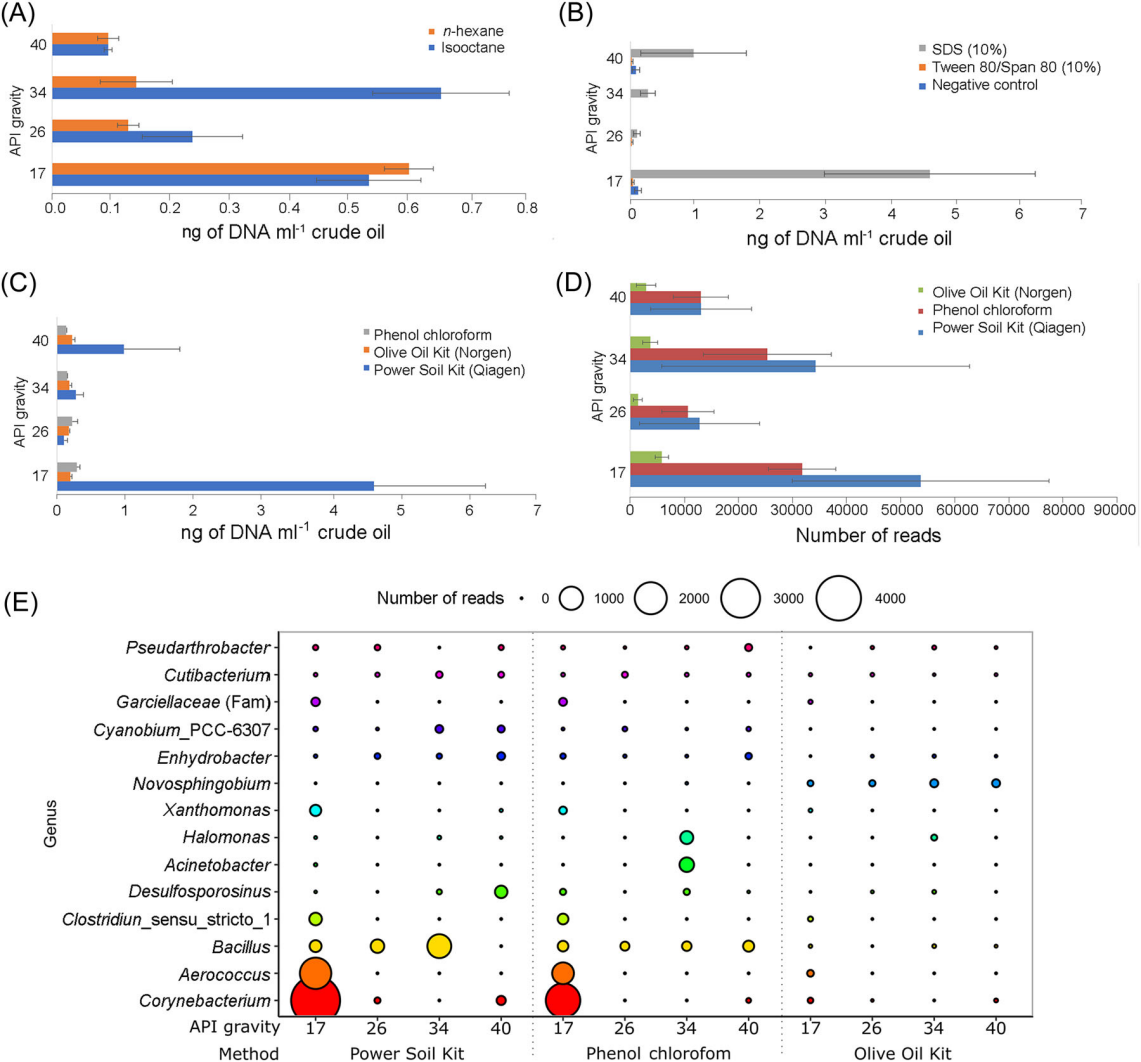
DNA yield in relation to the different used methods. (A) Isooctane versus *n*‐hexane. (B) Tween 80/Span 80 versus SDS 10% surfactants. (C) Phenol–chloroform versus Olive Oil Kit versus PowerSoil Kit DNA extraction methods. (D) The number of reads obtained by the different extraction methods. Error bars indicate SE (*n* *=* 3). (E) Bubble plot of the number of reads of the 14 most abundant genera (*n* *=* 3). SDS, sodium dodecyl sulfate.

The method of Yoshida et al.^26^ uses isooctane as a solvent, but the study of Lappé and Kallmeyer^23^ suggested *n*‐hexane and methanol as alternatives. However, methanol is hydrophilic, and this excluded its use for DNA extraction. We, therefore, tested *n*‐hexane and isooctane. In most cases, isooctane was the solvent that provided the highest DNA yield (Figure 2A). However, for API 17 oil, that is, the most biodegraded oil in this study, *n*‐hexane performed marginally better but still within 1 standard deviation. For API 40 oil, both solvents had the same yield. Due to the nature of the oils and the necessity to obtain a pellet to extract DNA from, we could not carry out a negative control with no solvent. All subsequent tests were carried out with isooctane.

### Surfactant test

After the initial solvent step was implemented, we assessed whether the use of surfactants increases the DNA yield. The combined nonionic/ionic surfactants (Tween 80/Span 80) performed worse than the negative control with no surfactant (Figure 2B). SDS was the surfactant that delivered the highest DNA yield, especially when combined with a heating step at 65°C for 10 min. This combination (SDS plus heating) resulted in an up to 10‐fold increase in DNA yields when compared to a pretreatment that only uses a solvent (Figure 2A, B). The heating step (10 min at 65°C) was adopted from the Norgen Olive Oil DNA extraction kit described in the “extraction test” section. Such a step is also mentioned in the literature, recommending 65°C but with different exposure time^37^, ^38^. We carried out preliminary tests that confirmed that this step results in higher DNA yields, and we, therefore, applied it to all subsequent extractions.

The amount of surfactant added to the bead tubes was based on literature values^39^, ^40^ and fine‐tuned in a serial dilution experiment. We obtained the best results by using a final concentration of 0.33% surfactant, corresponding to 50 µl of surfactant at 10% concentration for a 2‐ml bead tube mix containing approximately 1.5 ml of oil pellet and extraction reagents.

The Tween 80/Span 80 mix was prepared according to the hydrophile–lipophile balance (HLB) systems manual^31^ to obtain a water‐in‐oil emulsion with an HLB value of 10.7. This value was chosen based on the average oil pellet volume and the volume of the reagents added in the bead tubes for DNA extraction. This meant that, for a blend of 60% Tween 80 and 40% Span 80, 6 ml of Tween 80 and 4 ml of Span 80 were pipetted into 90 ml of deionized water to obtain a 10% solution. Due to the high viscosity of the Span 80 and Tween 80, the tips of the pipettes were cut off. The emulsion was achieved during the bead‐beating process at the beginning of the DNA extraction procedure.

### Extraction method test

We tested two commercial DNA extraction kits (DNeasy PowerSoil Pro Kit, Qiagen; Olive Oil Kit, Norgen) and phenol–chloroform extraction^39^ (Figure 2C). The samples were pretreated using isooctane as a solvent and SDS as a surfactant as these steps were shown to be effective in increasing the DNA yield. After the addition of SDS to the bead tubes, the samples were processed according to the manufacturer's recommendations.

The DNeasy PowerSoil Pro Kit (Qiagen) produced the highest yield. However, some of the DNA extracts prepared with this kit showed a milky coloration, and PCR often did not work without further cleaning steps.

However, we could solve this issue by cooling the DNA extract to 4°C and centrifuging the reaction tube at 11,000*g* for 1 min. After centrifugation, the pellet was discarded, and the supernatant could be used for PCR amplification.

Phenol–chloroform extraction often failed for the API gravity 26 oil. In some cases, after the addition of phenol–chloroform and the centrifugation step, the lysate remained mixed with the oil and did not separate. This was most probably due to a similar density of some compounds present in the oil pellet and the lysate. We were not able to identify a definitive cause of this phenomenon as it occurred randomly. We resolved the issue by processing the API 26 oil with more replicates to obtain three suitable replicate extracts.

### 16S rRNA

The number of reads obtained by 16S rRNA sequencing reflects, to some degree, the DNA extraction yield, but we did not find any correlation between the number of reads and API gravity (Figure 2D).

The PowerSoil Pro Kit (Qiagen) appeared to be the most effective, with more than 50,000 reads on average for oil with API gravity 17 (Figure 2D).

The low read numbers in some of the treatments (Figure 2D), especially with the Olive Oil Kit, hampered the comparability between the methods. The sequencing results (Figure 2E) of the test samples showed contaminants of potentially human origin and aerobic organisms that clearly did not originate from the anoxic oil reservoir. The detection of contaminants in the test samples is consistent with the lack of proper handling precautions required for microbiological analysis, such as sterile sampling equipment and anaerobic storage, which occurred before the samples reached our laboratory. The negative controls, however, showed that these contaminants were not introduced during the extraction procedure in our lab. The obvious contaminants, that is, the genera that have been reported in association with humans but never with hydrocarbons, were manually removed from the sequencing results (Figure 2E).

### Application of the new method

We applied the described method to pristine crude oil samples from three different reservoirs with API gravity in the range of 36–38 (P1, P2, and P3). We consider these oils to be pristine as no seawater flushing for enhanced oil recovery has been carried out. With seawater flushing, marine microorganisms are introduced into the oil reservoir and potentially overprint the indigenous microbial community due to the much greater population density in seawater^41^.

The samples were collected aseptically and stored under anaerobic conditions in the dark at 4°C and sample processing was conducted in an anaerobic glovebox. Due to their pristine conditions, the oils are expected to have low cell numbers in the order of 10^1^ to 10^3^ ml^−1^ of oil. As low cell density also means low DNA concentrations, we processed 25 ml of oil per sample. The resulting pellet was too large to fit into a single DNeasy PowerSoil Pro Kit (Qiagen) bead tube and therefore we used the DNeasy PowerMax Soil Kit (Qiagen). The kit is equivalent to the DNeasy PowerSoil Pro (Qiagen) but designed for larger volumes of material. Even though the oil volume used was constant, oil pellets obtained after the isooctane treatment varied considerably between a few microliters and 7–8 ml.

**Figure 3:**
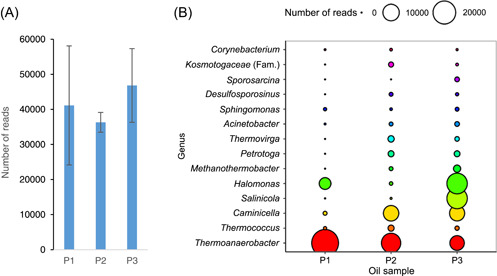
Sequencing results from pristine crude oil samples. (A) Reads from the pristine crude oil samples. DNA concentrations in all pristine samples were 0.011 (±0.001) ng/ml of crude oil. Error bars correspond to SE (*n* = 3). (B) Plot of the 16 most abundant genera from the pristine crude oil samples (*n* = 3). P1, P2, and P3, pristine oils originating from different oil reservoirs.

All samples provided a sufficiently high number of 16S rRNA sequencing reads (Figure 3A).

Considering that most of the taxa found (Figure 3B) are anaerobic, halotolerant, and thermophilic, we can deduce that what we observe is the indigenous community of the reservoirs. We could furthermore observe an overall similarity of the genera found in the different samples but with a distinct local signature of each single well.

## DISCUSSION

To the best of our knowledge, so far, there have been no published data about DNA yields from crude oil. This lack of available data poses a challenge in evaluating our DNA yield.

We, therefore, assessed our DNA extraction efficiency by calculating the cell density of our samples by the DNA yield obtained by our samples. To do so, we calculated the amount of DNA per cell using the formula below and divided the amount of obtained DNA by the per‐cell DNA weight.

$$\text{DNA}\;\;\text{weight}p\text{er}\mathrm{cell}\;=\left(\text{Genome}\;\text{size}\times 618\right)/\left(6.022\times 10^{23}\right)$$

where for genome size, we used the number of base pairs of the genome of the most abundant taxon of each sample, data taken from the JGI database; 618 represents the average molar mass of a base pair in bound form (in g/mol); and 6.022 × 10^23^ is Avogadro's constant.

With this method, we could obtain an approximate number of cells per milliliter of crude oil.

We compared the obtained numbers (Table 2) with previously reported cell abundances in oil reservoirs^42^, ^43^ and found that our approximation is well in line with the literature data, which in turn suggests that our DNA extraction method performed well. The calculated cell abundance reflects well the expected states of the oils: pristine samples (P) from high‐temperature reservoirs (76–80°C) show cell abundance in the range of 10^3^ cells/ml of oil, whereas the test samples (T), exposed to oxygen and not handled for microbiological purposes, which lead to secondary growth, had 10^4^–10^6^ cells/ml of crude oil.

**Table 2:** Approximate cell abundance calculated via genome size and DNA yield.

Oil sample	T1	T2	T3	T4	P1	P2	P3
Reference genome	*Corynebacterium* spp.	*Bacillus* spp.	*Bacillus* spp.	*Corynebacterium* spp.	*Thermoanerobacter* spp.	*Thermoanerobacter* spp.	*Thermoanerobacter* spp.
The average number of bp	2.6 Mbp	4.9 Mbp	4.9 Mbp	2.6 Mbp	2.4 Mbp	2.4 Mbp	2.4 Mbp
Obtained DNA yield (ng/ml) of crude oil	4.61	0.1	0.27	0.97	0.012	0.011	0.01
Number of cells/ml of crude oil	1.7 × 10^6^	2 × 10^4^	5.3 × 10^4^	3.6 × 10^5^	4.8 × 10^3^	4.4 × 10^3^	4 × 10^3^

T1, T2, T3, and T4, test oils of different API gravities; P1, P2, and P3, pristine oils originating from different oil reservoirs.

Contrary to our hypothesis, DNA yield did not correlate with API gravity. In most tests, the API 26 oil yielded the lowest DNA concentrations (Figure 2A–C). The 16S rRNA sequencing results showed the same trend, with the number of genomic reads being the lowest for API 26 oil (Figure 2D). The reason for this difference could be due to the amount and consistency of pelleted material deposited at the bottom of the reaction tube after the isooctane treatment. The API gravity 26 oil produced the visibly largest pellet, which had a tar‐like appearance and appeared to be more hydrophobic than the pellets from the other oils. The hydrophobicity might have hindered the access of the reagents to the DNA in the pellet and therefore reduced the extraction yield.

Because the oils used for the method development with different origins, were not sampled aseptically, and were exposed to oxygen, we were not able to assess whether there is a correlation between microbial community and the level of biodegradation.

From the results of the solvent test, we could observe that both isooctane and *n*‐hexane are generally suitable solvents for pre‐extracting the aqueous phase from the oil, with isooctane performing marginally better. Lappé and Kallmeyer^23^ also noted the suitability of *n*‐octane, an isomer of isooctane, and *n*‐hexane as useful solvents for hydrocarbon removal from soil‐contaminated samples. During our preliminary experiments, we also tested mixtures of *n*‐hexane and isooctane. The mixtures, however, performed worse than the separated reagents and were therefore not taken into consideration. For further studies, we suggest testing both isooctane and *n*‐hexane, especially for oils with lower API gravity, to assess which solvent performs best.

During preliminary evaluations, we explored various ratios of oil and solvent. Our observations indicated that the ratio could be slightly adjusted up to 4:6 (isooctane–oil) for higher API gravity oils, enabling the processing of larger oil quantities in a single extraction. However, exceeding this ratio resulted in the coextraction of oil material that posed challenges for PCR amplification. Lower API gravity oils^17^, ^18^, ^19^, ^20^, ^21^, ^22^, ^23^, ^24^, ^25^, ^26^ performed better at a 1:1 ratio. To ensure standardization, we maintained the oil–solvent ratio at 1:1 in all our tests.

During the isooctane step, we often noticed that the mix of crude oil and isooctane leaked from the screw‐cap reaction tubes in the centrifuge during spinning. This issue often led to a laborious cleaning of the centrifuge to remove crude oil traces from the rotor and the chamber using isooctane, as normal cleaning solvents like 70% ethanol or isopropanol do not work. The use of centrifuge tubes with rubber seal caps did not overcome the issue. We solved the problem by placing a thin layer of paper towel over the rotor and piercing it with the tubes while inserting them into the rotor.

Although the nonionic surfactant mix formed a visibly uniform emulsion with the pellet from the solvent pretreatment, the DNA extraction with the Tween 80/Span 80 blend did not produce sufficient DNA yields (Figure 2B). We assumed that the emulsion reduces the droplet size of the oily material by creating a uniform mix of aqueous and oily phases^44^, ^45^, so the cells within the emulsion could be easily separated by centrifugation. However, the emulsion very likely caused the opposite effect because the emulsion has a higher apparent viscosity as compared to the separated aqueous and oil phase^46^. Most likely, the cells and the beads in the bead tube were immobilized and this might have prevented the cell lysing process from proceeding. As a result, the nonionic surfactant mix performed worse than the control without surfactant addition.

SDS, on the contrary, increased the DNA yield by up to an order of magnitude when compared to the control without surfactant. Prior studies already confirmed SDS as the “go‐to” surface active agent when working with crude oils. Sharma et al.^47^ proved the capability of SDS in decreasing the viscosity of biodegraded crude oil and Hosnani et al. and Urum et al.^48^, ^49^ proved the effectivity of SDS in removing crude oil from contaminated soils.

Temperature has an important effect on the behavior of surfactants^32^. Surfactants in general^29^ and specifically SDS^49^ have higher efficiency at temperatures around 50–60°C. A temperature of 65°C was cited in the literature for SDS‐based DNA extraction from seafloor sediments^50^ and for DNA extractions from olive oil^37^, ^38^. For our crude oil samples, we found that at 65°C we could sufficiently reduce the viscosity of the oil pellet in the bead tube and obtain sufficient DNA. For practical reasons, we did not investigate a wider temperature spectrum and we recommend further investigations to tweak the temperature and time of exposure of the oil pellets using SDS and the PowerSoil Pro Kit reagents.

One of the drawbacks of SDS is that it is most likely carried through DNA extraction and hinders amplification and therefore requires an additional cleanup step. However, we observed that by cooling the samples to 4°C and storing them for a week, SDS precipitated, as well as most likely other inhibitors present in the oils. After some tests, we noted that the precipitation process could be speeded up by storing the eluted DNA at 4°C overnight and centrifuging it for 1 min at 11,000*g*. This step was sufficient to obtain amplifiable DNA.

The PowerSoil Pro Kit provided the highest DNA yields and gave the highest number of reads in the 16S rRNA sequencing. Furthermore, the PowerSoil Pro Kit does not expose the operator to harmful substances compared to phenol–chloroform. However, phenol–chloroform extraction seems to offer a valid and financially more viable option for DNA extraction from crude oil as long as the lysate can be separated by centrifugation.

We chose the DNeasy PowerSoil Pro Kit (Qiagen) because it is a standard in environmental microbiology, and the earlier version of the kit, PowerSoil, has been used previously for DNA extraction from crude oils^36^ as well as from sediments and soils associated with hydrocarbons^51^, ^52^, ^53^, ^54^. The PowerSoil Pro Kit features fine beads (0.1 mm) for bead beating and it uses a proprietary “inhibitor removal technology” within one of the reagents, apparently capable of removing organic inhibitors commonly found in environmental samples.

As a second commercial kit, we chose the Olive Oil DNA Isolation Kit (Norgen) as it was the only kit on the market that we could find that is specifically designed for hydrophobic substrates. To our knowledge, it has not been tested previously on crude oil samples.

The reagents of the Olive Oil Kit are proprietary, and they are labeled as lysis buffer L, binding buffer B, wash solution A, and elution buffer B. The kit does not use a bead‐beating step but includes an incubation step of 10 min at 65°C with the lysis buffer and DNA‐binding spin columns for the washing step.

As for the 16S rRNA results from the oils used for method development, there are discrepancies in the taxa found between the extraction techniques (Figure 2E). The results seem replicable between extraction methods only when the number of reads is above 20,000, such as in API 17 oil. Lab handling contamination can be ruled out by negative controls. Hence, these discrepancies are probably due to the low number of reads obtained from DNA extracts from the oils with API gravities 26, 34, and 40, using the phenol–chloroform method and the Olive Oil Kit.

With the test oils, we were limited by the volume of oil available from each API gravity and wanted to ensure enough replicates; we, therefore, used 3 ml of crude oil for each replicate for our methodological tests. Furthermore, the pellet produced from 3 ml of oil is small enough to fit into a 2‐ml DNeasy PowerSoil Pro Kit bead beating tube.

In the method application, we used 25 ml of oil in combination with the large‐volume DNeasy PowerMax Soil Kit (Qiagen) to ensure enough DNA. All samples had read numbers between 30,000 and 55,000 (triplicate average). We recommend that future studies use oil volumes of at least 25 ml for obtaining a better picture of the oil microbiome. The pristine oil samples analyzed in this study contained multiple taxa, namely, *Thermoanaerobacter, Petrotoga, Kosmotoga, Thermovirga*, and *Thermococcus*. These genera are frequently observed in high‐temperature pristine oil reservoirs^15^, ^16^.

Although the Olive Oil Kit is the only kit for DNA extraction from nonpolar substances that we could find on the market, it did not provide satisfactory yields with crude oil samples (Figure 2C–E). This was probably due to the lack of beads in the beating step and the hard consistency of the tar‐like crude oil pellet obtained after the isooctane pretreatment.

In this study, we present a new method that effectively extracts DNA from crude oils with a wide range of API gravities. The extracted DNA is sufficiently clean for subsequent amplification or sequencing. However, crude oil is a complex matrix composed of a multitude of hydrocarbons, and the method described here requires fine‐tuning to a specific oil sample. Furthermore, as the range of combinations of surfactants, solvents, extraction methods, and API gravity oils is potentially endless, future investigations should explore this further.

## MATERIALS AND METHODS

### Samples

The oils used for this study were provided by Aker BP, Lysaker, Norway, and come from different oil fields in the Barents Sea and North Sea and cover a wide range of API gravity, from 17 to 40. We had two types of samples:

- The first type had not been intended for microbiological analyses and therefore no precautions for sterility or anoxic storage were taken during field sampling.
- The second type had been sampled specifically for microbiological purposes, that is, sterilized glass bottles filled without headspace to avoid oxygen contamination. According to the oil field operators, these fields can be considered pristine, as no seawater injections have been carried out for extracting the oil.

All the oils were stored in glass bottles at 4°C upon delivery at GFZ Potsdam.

The first type of samples had air headspace, and all handling was carried out in a fume hood to avoid exposure to volatile hydrocarbons. We named the test oils T1, T2, T3, and T4. The second type of oils was sampled in sterile bottles, kept strictly anaerobic, and the subsampling was carried out in an anaerobic glovebox. We named these pristine oil samples P1, P2, and P3.

Once in the reaction tubes, subsequent sample handling of both types of samples was carried out in a fume hood. Recovery of oil pellets and DNA extraction were carried out in a laminar flow cabinet with flamed tools.

All DNA extractions have been carried out in an S2 lab with positive pressure and filtered air.

Our crude oil samples often showed small droplets of probably brine water on the walls of the bottles. To distribute the droplets uniformly, we manually shook the bottles before pouring 3 ml of the content into 15‐ml reaction tubes. Each experiment was carried out in triplicate and with negative controls.

### Reagents and materials

- Surfactants: SDS 98% (Sigma), Tween 80 (AppliChem), and Span 80 (Sigma‐Aldrich).
- Solvents: Isooctane (2,2,4‐trimethylpentane) and *n*‐hexane.
- Commercial kits for DNA extraction: PowerSoil Pro Kit (Qiagen), PowerMax Soil Kit (Qiagen), and Olive Oil Kit (Norgen).
- Reagents for phenol–chloroform extraction: cetrimonium bromide (CTAB); phenol–chloroform–isoamyl alcohol (25:24:1); guanidine hydrochloride (GuaHCl) solution (6 M of GuaHCl 1× TE buffer [pH 6.7], 10 mM of Tris–HCl, and 1 mM of EDTA and sterile filtered); and washing buffer composed of 50% EtOH, 125 mM of NaCl, 10 mM of Tris, and 1 mM of EDTA.
- Qubit 2.0 fluorimeter with double‐strain high‐sensitivity (dsDNA HS) assay kit containing buffer and fluorescent dye (Invitrogen).
- Reagents for PCR: OptiTaq DNA polymerase (EurX), bovine serum albumin (BSA), 10× Pol buffer (EurX), dNTP (EurX), MgCl_2_ (EurX), PCR‐grade water (EurX), and barcoded 515F 806R primers.
- Reagents for post‐PCR cleanup: AMpure beads, ethanol 70%, and elution buffer.
- Centrifuges: Sigma 6‐16KS and Thermo Scientific Fresco 17.
- Bead beating: Vortex Genie 2 with horizontal adapter and FastPrep (MPI).
- 15‐ml Falcon tubes (VWR)
- Spatulas and spoons

### Solvent test

The isooctane method requires mixing isooctane and crude oil in a 1:1 ratio, leaving the samples overnight at 4°C, followed by spinning at 5000*g* for 1 h, discarding the supernatant, resuspending the pellet in the initial volume of isooctane, spinning again at 5000*g* for 30 min, discarding the supernatant, and adding the pellet to the bead tubes for DNA extraction.

The pellets obtained after centrifugation were transferred to the PowerSoil Pro bead tubes for DNA extraction. DNA was extracted according to the manufacturer's protocol. At this point, DNA extraction was already possible but the yields were low. Further improvement of the DNA yield was needed, and preliminary work showed the effectiveness of the addition of surfactant to the bead tubes before DNA extraction.

### Surfactants test

We tested a blend of Tween 80/Span 80 and SDS. The pellet from the isooctane extraction and 50 µl of the surfactants were added directly to the bead tubes. The bead tubes were briefly vortexed and heated for 10 min at 65°C to allow the surfactants to act more efficiently and to melt the oily material. From this point on, we followed the manufacturer's standard kit protocols and the phenol–chloroform extraction method according to Nercessian et al^39^.

### Extraction test

Once we verified the best solvent and surfactant for our purpose, we assessed the different DNA extraction methods. For the extraction test, we processed the oil samples with isooctane according to Yoshida et al.,^26^ pelleted the oils via centrifugation, and added the pelleted material to the bead tube. We then added SDS to the bead tubes, heated for 10 min at 65°C, and from this point on, we followed the manufacturer's protocols and the phenol–chloroform DNA extraction according to Nercessian et al^39^. We assessed the efficiency of the extraction methods by measuring the resulting DNA yields and by the number of reads and taxonomical results obtained using 16S rRNA sequencing.

The phenol–chloroform extraction method is a modified version of a protocol from Nercessian et al^39^. A crude oil pellet (up to 0.5 g) was added to a 2‐ml screw cap tube filled 1/3 with zirconia beads and glass beads of different sizes (0.1–1 mm diameter). We then added 0.6 ml of cetrimonium bromide (CTAB) buffer, 110 µl of SDS 10%, and 0.6 ml of phenol–chloroform–isoamyl alcohol (25:24:1) to the screw cap tube and the tube was shaken in a bead beater (FastPrep, MPI) for 45 s at 6 m/s. The bead tubes were centrifuged at 16,000*g* at 4°C for 10 min and the upper phase was pipetted to a new reaction tube to which an equal volume of chloroform–isoamyl alcohol (24:1) was added. The reaction tubes were centrifuged at 16,000*g* at 4°C for 10 min and 0.5 ml of the supernatant was transferred to a new reaction tube together with 1 ml of guanidine hydrochloride (GuaHCl) solution.

A Zymo‐Spin IIICG Silica‐based spin column (Zymo Research) was inserted into the 2‐ml reaction tube and 0.6 ml of the solution was added to the column. Afterward, the tube was spun at 5000*g* for 1 min, and the reagents that flowed through were discarded. This step was repeated until the solution had completely passed through the spin column. Then, we added 0.5 ml of washing buffer (50% EtOH, 125 mM of NaCl, 10 mM of Tris, and 1 mM of EDTA) to the spin column, and the solution that flowed through was discarded.

The spin column was transferred to a clean reaction tube and 60 µl of PCR‐grade water was added to the column and incubated for 10 min. The reaction tube with the spin column was then centrifuged at 5000*g* for 1 min and the spin column was discarded. The solution remaining in the reaction tube is the eluted DNA.

### DNA quantification

We quantified DNA using the Qubit 2.0 device following the dsDNA HS assay.

### 16S rRNA sequencing and data processing

Bacterial and archaeal 16S rRNA gene fragments were PCR amplified in triplicate primers 515F (5′‐GTGTGYCAGCMGCCGCGGTAA‐3′) and 806R (5′‐CCGGACTACNVGGGTWTCTAAT‐3′). Primer pairs, including specific barcodes, were assigned to each of the PCR products to identify each sample from the pooled library after sequencing.

The final volume of the reaction mixture was 50 µl, containing 4 µl of DNA template, 0.5 µl of Taq DNA polymerase, 2 µl of dNTP mix and MgCl_2_, 5 µl of 10× Pol buffer C, 0.5 µl of BSA, 2.5 µl of primers, and 33.5 µl of PCR grade water.

PCR amplification was carried out using 5 min of initial denaturation at 95°C, followed by 32 cycles of 30 s at 95°C, 30 s at 56°C, and 1 min at 72°C and a final extension step of 72°C for 7 min.

The PCR product was cleaned using AMPure magnetic beads (Beckman Coulter Life Sciences). All the PCR products were pooled equally in a final concentration of 20 ng for paired‐end sequencing (2 × 300 bp) on Illumina MiSeq (Eurofins Genomics Europe Sequencing GmbH).

The sequencing library was demultiplexed using cutadapt v3.4^55^ using the following parameters: ‐e 0.2 ‐q 15,15 ‐m 150 ‐‐discard‐untrimmed identifying only read pairs with correct barcodes at both ends. The ASVs were generated using trimmed reads and the DADA2 package v1.20^56^ with R v4.1 using the pooled approach with the following parameters: truncLen=c(240,200), maxN=0, rm.phix=TRUE, minLen=200. Taxonomic assignment was done using DADA2 and the SILVA database v138^57^. Subsequently, ASVs representing chloroplasts, mitochondria, and singletons were removed.

## AUTHOR CONTRIBUTIONS

**Armando Alibrandi**: Conceptualization (equal); data curation (equal); investigation (equal); methodology (equal); writing—original draft (equal); writing—review and editing (equal). **Rolando di Primio**: Funding acquisition (equal); resources (equal); writing—review and editing (equal). **Alexander Bartholomäus**: Data curation (equal); software (equal); writing—review and editing (equal). **Jens Kallmeyer**: Conceptualization (equal); funding acquisition (equal); project administration (equal); supervision (equal); validation (equal); writing—review and editing (equal).

## ETHICS STATEMENT

The study in this article did not involve any trials on humans or animals.

## CONFLICT OF INTERESTS

The authors declare no conflict of interests.

## Data Availability

The data that support the findings of this study are openly available in the European Nucleotide Archive (ENA) at EMBL‐EBI at https://www.ebi.ac.uk/ena/browser/view/PRJEB65234, reference number PRJEB65234.
